# Effects of Metal Doping on the Structure and Water–Gas Shift Reaction Performance of Copper–Manganese Catalysts

**DOI:** 10.3390/molecules31203630

**Published:** 2026-10-12

**Authors:** Fang Wu, Runxia He, Qianqian Hu, Gao Saishengtai

**Affiliations:** 1 Department of Chemical Engineering, Inner Mongolia Vocational College of Chemical Engineering, Hohhot 010070, China; nhyyjk2021@126.com (F.W.); hggsst@126.com (G.S.); 2 College of Chemical Engineering, Inner Mongolia University of Technology, Hohhot 010051, China; baodingjincan@163.com

**Keywords:** water–gas shift reaction (WGSR), Cu-Mn catalyst, metal doping, Zr modification, structure–performance relationship

## Abstract

Cu-Mn catalysts modified with Zr, Ce, Fe, Al, and Zn were prepared by co-precipitation and evaluated for the water–gas shift reaction (WGSR) using a common conditioning and cooling protocol. Among the tested compositions, the 5 mol% Zr sample gave the highest reported mean CO conversion at 200 °C, 60.1%, compared with 23.1% for undoped CuMn. XRD, BET, H_2_-TPR, NH_3_-TPD, N_2_O-assisted surface reduction (s-TPR), and XPS were used to compare phase composition and surface properties. Within the Zr series, the 5 mol% sample also had the largest model-derived Cu surface area per catalyst mass, 44.1 m^2^ g^−1^, whereas the apparent fractional Cu dispersion was highest at 8 mol% Zr. CO conversion did not increase monotonically with Zr loading, and the higher-conversion 5 mol% sample had larger spent Cu crystallites than undoped CuMn. These comparisons distinguish nominal loading, fractional dispersion, and mass-specific Cu area as separate descriptors when relating composition to WGSR performance. Mechanistic interpretations and long-term durability require further evidence.

## 1. Introduction

The water–gas shift reaction (WGSR, CO + H_2_O ⇌ CO_2_ + H_2_) is used to enrich hydrogen and adjust the composition of synthesis gas. Catalyst performance depends on temperature, feed composition, and prior reaction history, and thermodynamic equilibrium must be considered when comparing CO conversion at different temperatures [1,2].

Gold-based catalysts provide one established approach to low-temperature WGSR [3]. The development of non-noble-metal alternatives has also motivated studies of Cu-Mn mixed oxides, including catalysts derived from spinel precursors and catalysts modified through precipitation conditions or additional metal components [4,5,6,7,8]. These studies establish a relevant Cu-Mn context for examining the relationships among composition, reducibility, surface properties, and activity.

Composition is a useful variable for comparative studies of Cu-Mn catalysts. Earlier work directly examined third-metal effects in Cu-Mn mixed oxides [5], while ZrO_2_ modification has also been investigated in a CuO/Fe_2_O_3_ WGSR system [9]. Studies of CuO-CeO_2_ further consider structural and acid–base properties alongside WGSR activity [10,11]. These related systems provide context, but do not establish a mechanism for the present Cu-Mn catalysts. This work compares several dopants introduced by a common preparation route and then examines whether increasing the nominal Zr content produces a monotonic change in low-temperature conversion.

Cu-Mn catalysts modified with Zr, Ce, Fe, Al, and Zn were prepared using CuSO_4_·5H_2_O and MnSO_4_·H_2_O as the Cu and Mn precursors. Following the dopant comparison, a series containing 0, 2, 5, 8, and 20 mol% nominal Zr was evaluated. Activity was compared after the same conditioning and cooling program. The sample-specific experimental record is documented in Hu’s thesis [12], and the laboratory platform and common analytical methods are described in Zhi’s doctoral dissertation [13]. Structural observations, model-dependent surface descriptors, and mechanistic interpretations are distinguished below.

## 2. Results and Discussion

### 2.1. Effect of Metal Ion Type on Cu-Mn Catalysts

#### 2.1.1. Catalytic Performance

**Figure 1:**
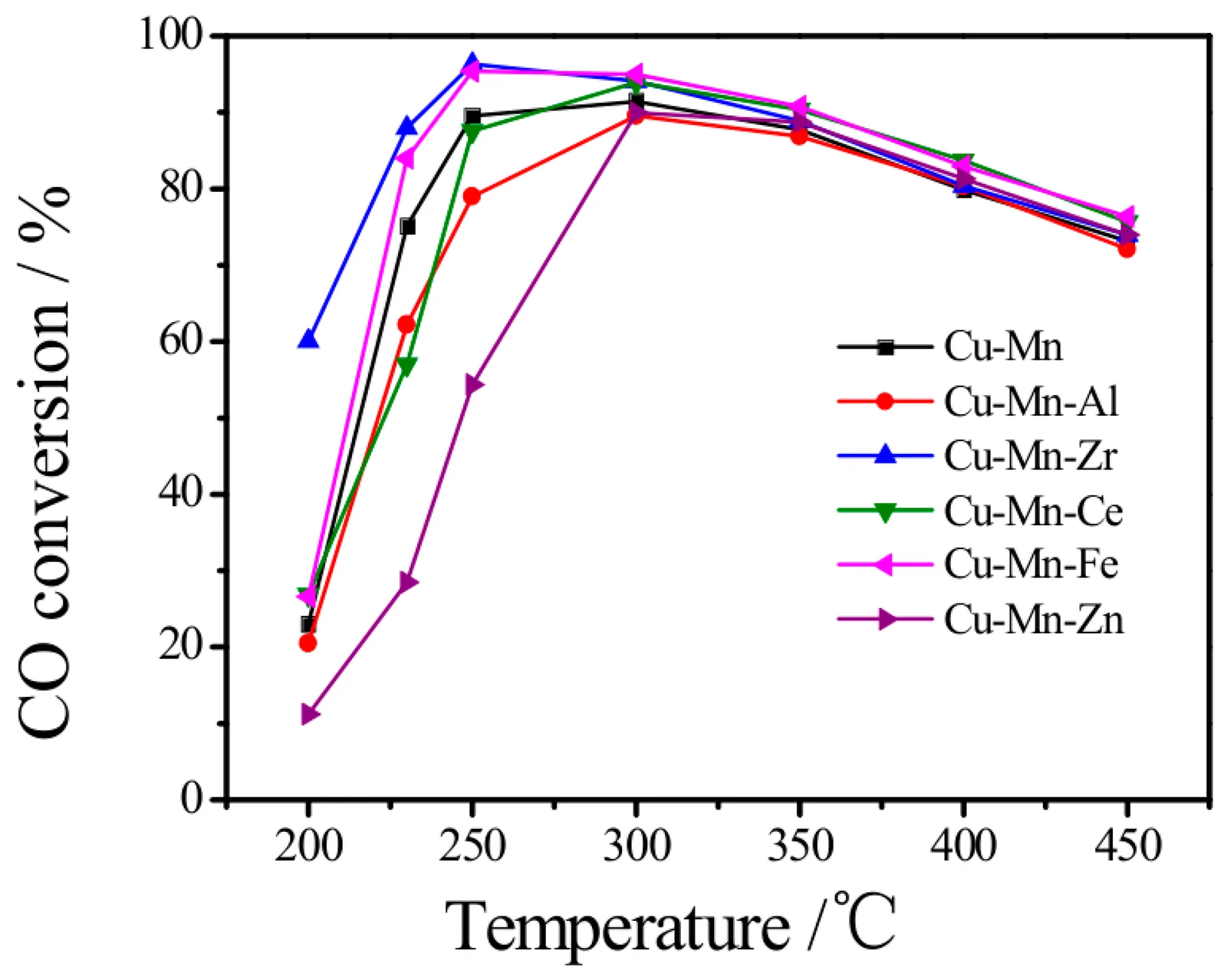
CO conversion of the different-metal series during cooling evaluations after the common 450 °C conditioning treatment. The graph is from Hu’s thesis, Figure 3-7(b) [12]. The intermediate evaluation temperature is 230 °C. Source legends use Cu-Mn for CuMn. The 450 °C conditioning histories are provided in Supplementary Figure S1.

Figure 1 compares the cooling evaluations following the common 450 °C conditioning treatment; the conditioning histories are provided in Supplementary Figure S1. At 200 °C, CuMn-Zr gives a reported mean CO conversion of 60.1%, compared with 23.1% for CuMn and 11.2% for CuMn-Zn [12]. CuMn-Zr reaches 96.4% at 250 °C. The dopant effect is therefore most clearly observed in the low-temperature response under this common reaction history.

The 200 °C conversions are well below the 99.0% equilibrium reference in Section 3.2, whereas the 96.4% conversion of CuMn-Zr at 250 °C is close to the calculated 97.4% value. Several active samples also approach the reference near 300 °C. It is therefore inappropriate to describe all results between 200 and 300 °C as far from equilibrium. At higher temperatures, convergence of the conversions is consistent with an increasing equilibrium constraint, but proximity must be assessed by sample and temperature. These comparisons do not establish intrinsic isothermal kinetic constants.

#### 2.1.2. Characterization of Cu-Mn Catalysts with Different Metal Dopants

##### XRD Analysis

**Figure 2:**
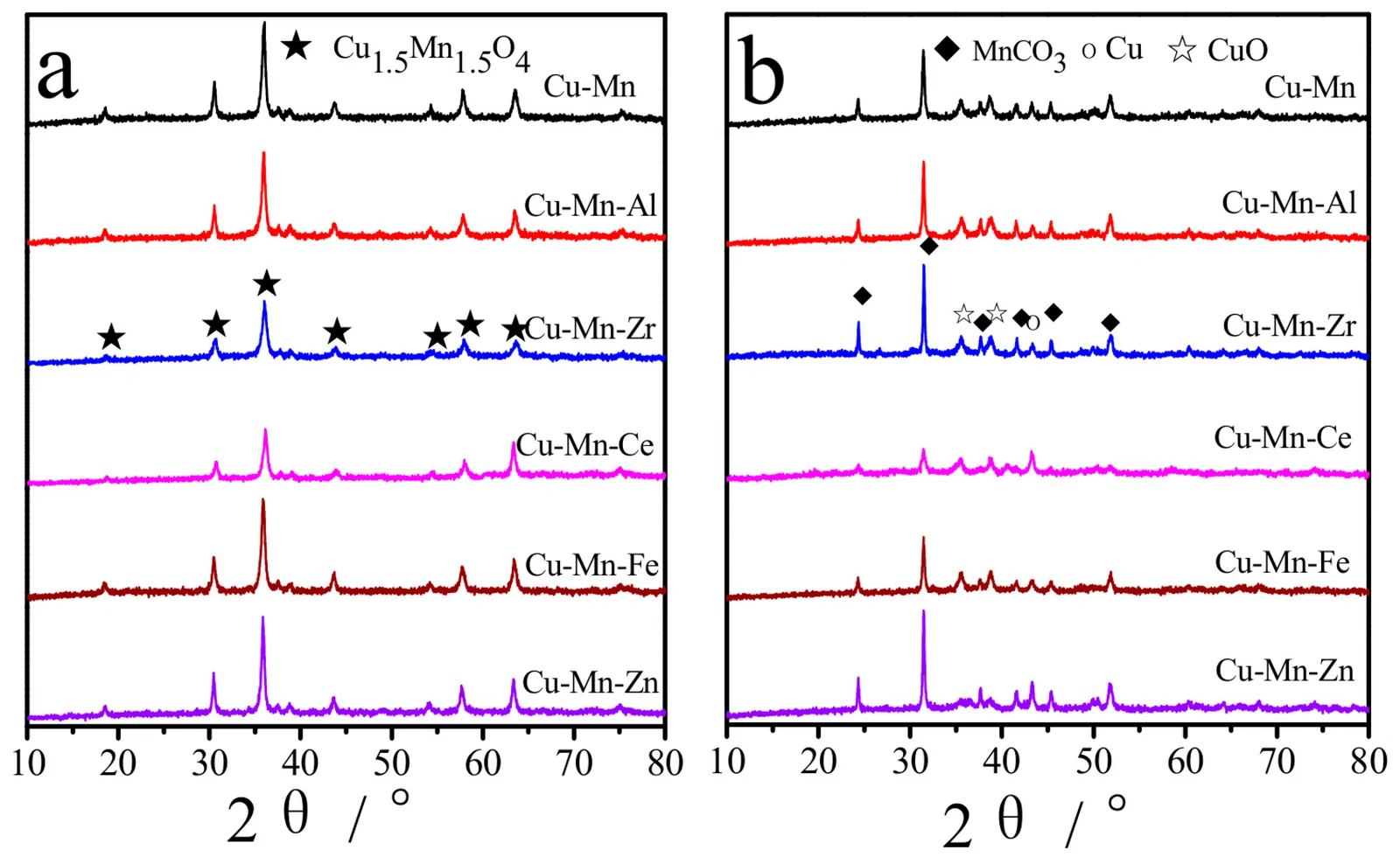
XRD patterns of the different-metal series: (**a**) calcined; (**b**) spent. Graphs and phase labels are from Hu’s thesis, Figure 3-8 [12].

Figure 2 shows XRD patterns before and after WGSR treatment. The calcined reflections were indexed mainly to a Cu-Mn spinel-type phase reported as Cu_1.5_Mn_1.5_O_4_ (JCPDS 35-1172) [12,14]. Diffraction matching alone does not determine exact cation valences, occupancies, or oxygen stoichiometry. For the nominal formula, assigning Cu an average oxidation state of +2 requires an average Mn oxidation state of +3.33: 1.5 × 2 + 1.5 × 3.33 ≈ 8. Mixed Mn^3+^/Mn^4+^ states can formally satisfy charge balance without requiring oxygen deficiency solely from the formula. This is a charge-counting example, not an experimental valence assignment. The spent patterns are indexed to MnCO_3_, CuO, and metallic Cu, indicating phase reconstruction. Changes in Cu and Mn oxidation state should therefore be considered in a reconstructed multiphase solid with coupled changes in oxygen and phase composition, rather than in an unchanged spinel lattice of fixed stoichiometry.

Table 1 combines the phase-specific XRD parameters and BET areas for the different-metal series. CuMn-Zr has a calcined spinel size of 15.6 nm, compared with 19.2 nm for CuMn. After reaction, its metallic Cu crystallite size is 50.3 nm, larger than the 30.4 nm value for CuMn. The better low-temperature conversion therefore cannot be attributed to smaller spent Cu crystallites. CuMn-Zr has the largest BET area among these samples in both the calcined and spent states, 52.1 and 41.7 m^2^ g^−1^. Small diffraction shifts describe differences in lattice spacing, but do not uniquely establish dopant substitution or spatially uniform cation dispersion.

##### H_2_-TPR/NH_3_-TPD/s-TPR Analysis

**Figure 3:**
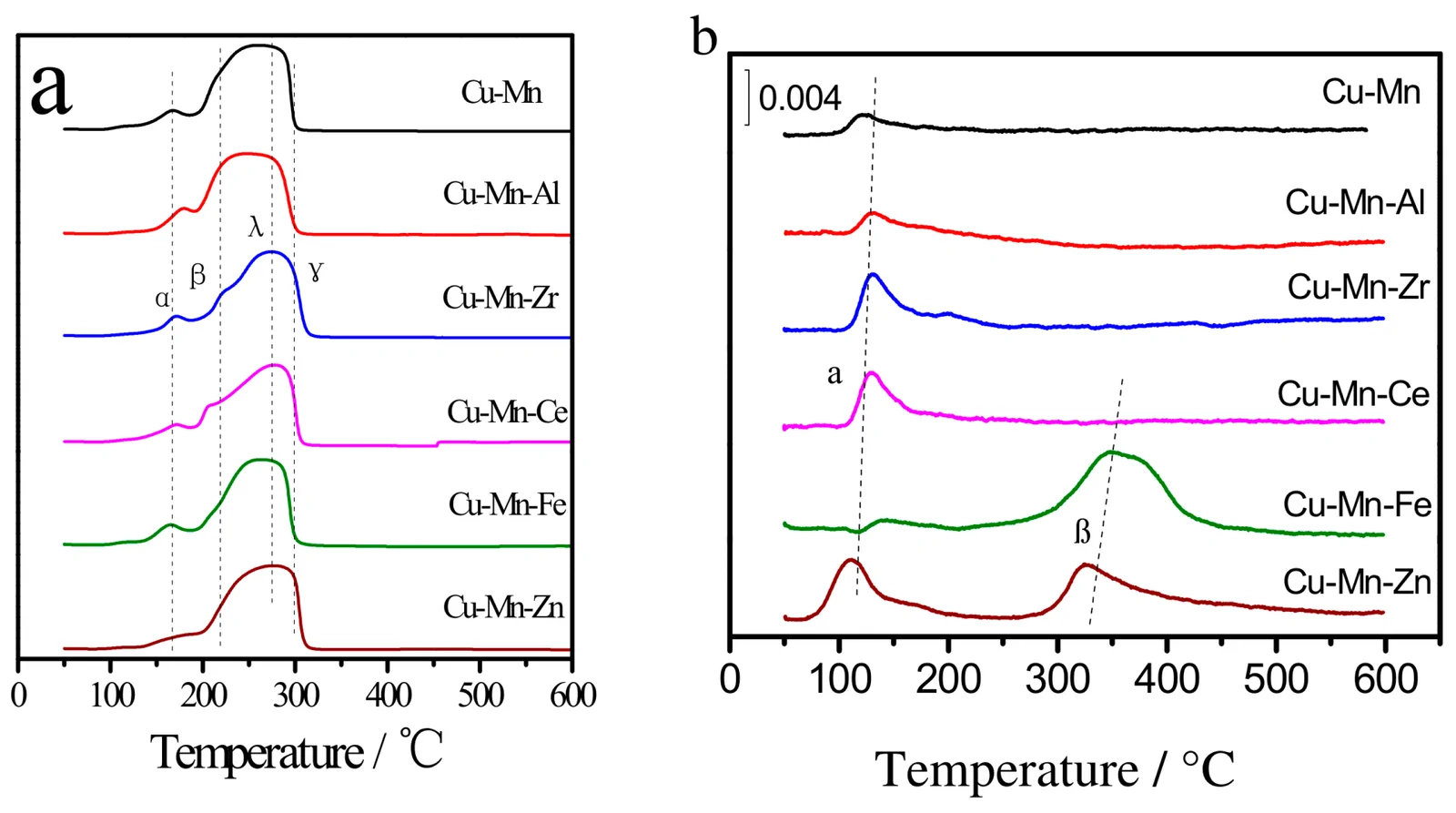
(**a**) H_2_-TPR and (**b**) NH_3_-TPD profiles of the different-metal series. Panels are from Hu’s thesis, Figures 3-12(a) and 3-14 [12]; the axis title in (**b**) has been typeset for clarity. Curve shapes and source peak annotations are retained.

Figure 3a shows the H_2_-TPR profiles. The source interpretation distinguishes CuO-related α features, Cu-Mn solid-solution β and λ features, and an overlapping Mn-related γ contribution [12,13]. Gaussian fitting is part of the shared-platform analysis, but peak labels alone do not demonstrate quantitative Cu/Mn separation for the s-TPR integrals. The reduction response changes with composition and contains overlapping contributions; it does not uniquely identify the operando active structure [15].

NH_3_ adsorption/desorption has been used to examine surface acidity in Cu-based WGSR catalysts [16]. In Figure 3b, the source profiles show low-temperature features near 100–130 °C and more pronounced higher-temperature features for Fe- and Zn-modified samples. Here they describe comparative NH_3_ retention and desorption under the stated pretreatment. They do not directly measure Cu dispersion or surface uniformity. Without calibrated uptake and complete gas parameters, no optimal acid-site density or causal acidity–activity relationship is inferred.

**Figure 4:**
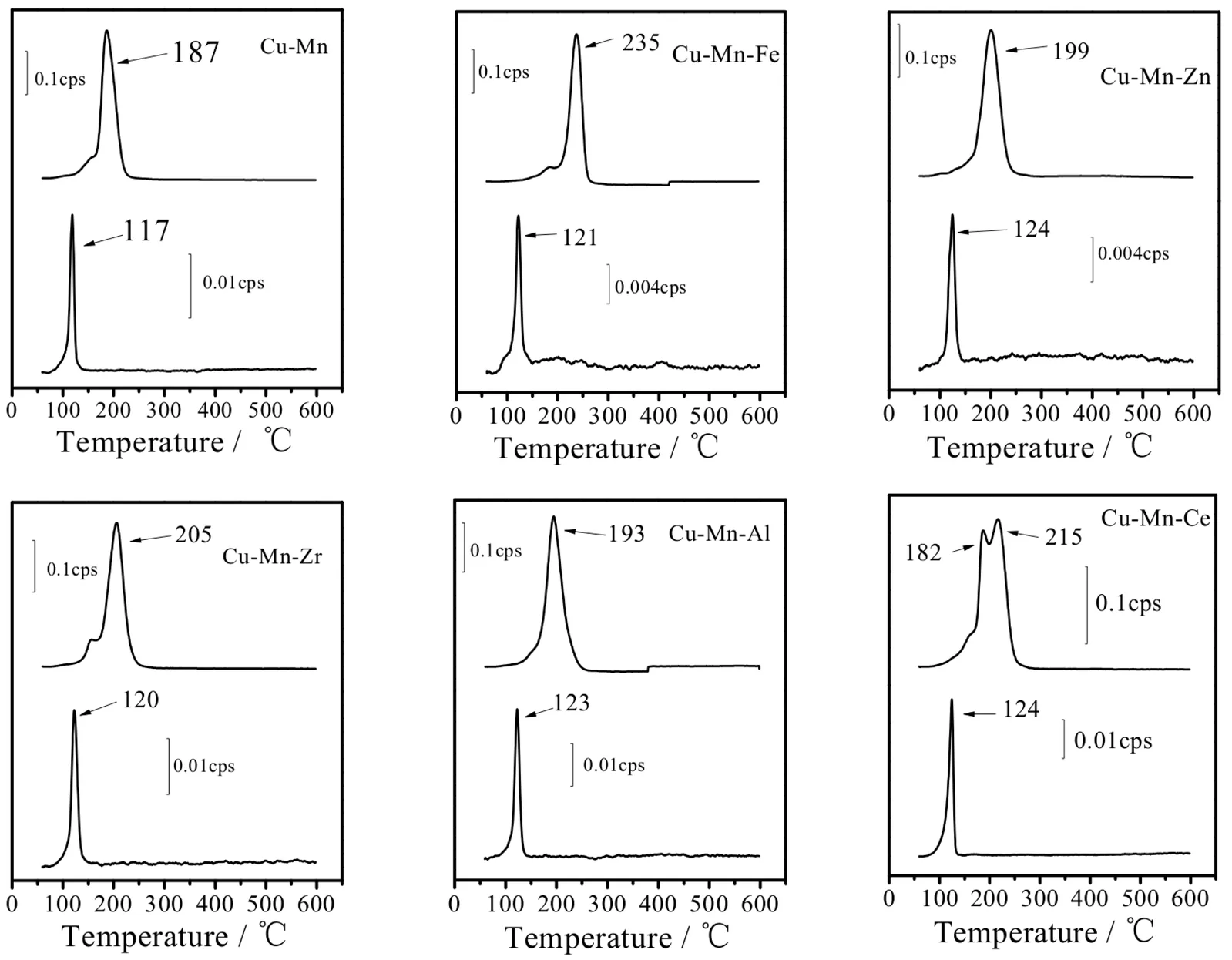
s-TPR profiles of the different-metal series from Hu’s thesis, Figure 3-13 [12]. Upper and lower traces in each panel are the first and second reductions, respectively. Original peak-temperature annotations are retained; they are not newly fitted values.

Figure 4 reproduces the source s-TPR profiles, while Table 2 lists the area inputs and uniformly recalculated descriptors. The calculated Cu surface area per unit catalyst mass increases from 21.3 m^2^ g^−1^ for CuMn to 44.1 m^2^ g^−1^ for CuMn-Zr; CuMn-Zn gives 32.5 m^2^ g^−1^. The second-stage profiles contain lower-temperature reduction features, consistent with the adopted surface oxidation–reduction procedure. Nevertheless, the estimates remain subject to the Cu reduction and selective oxidation assumptions discussed in Section 3.3. Fractional dispersion and mass-specific area are reported as separate quantities.

##### XPS Analysis

**Figure 5:**
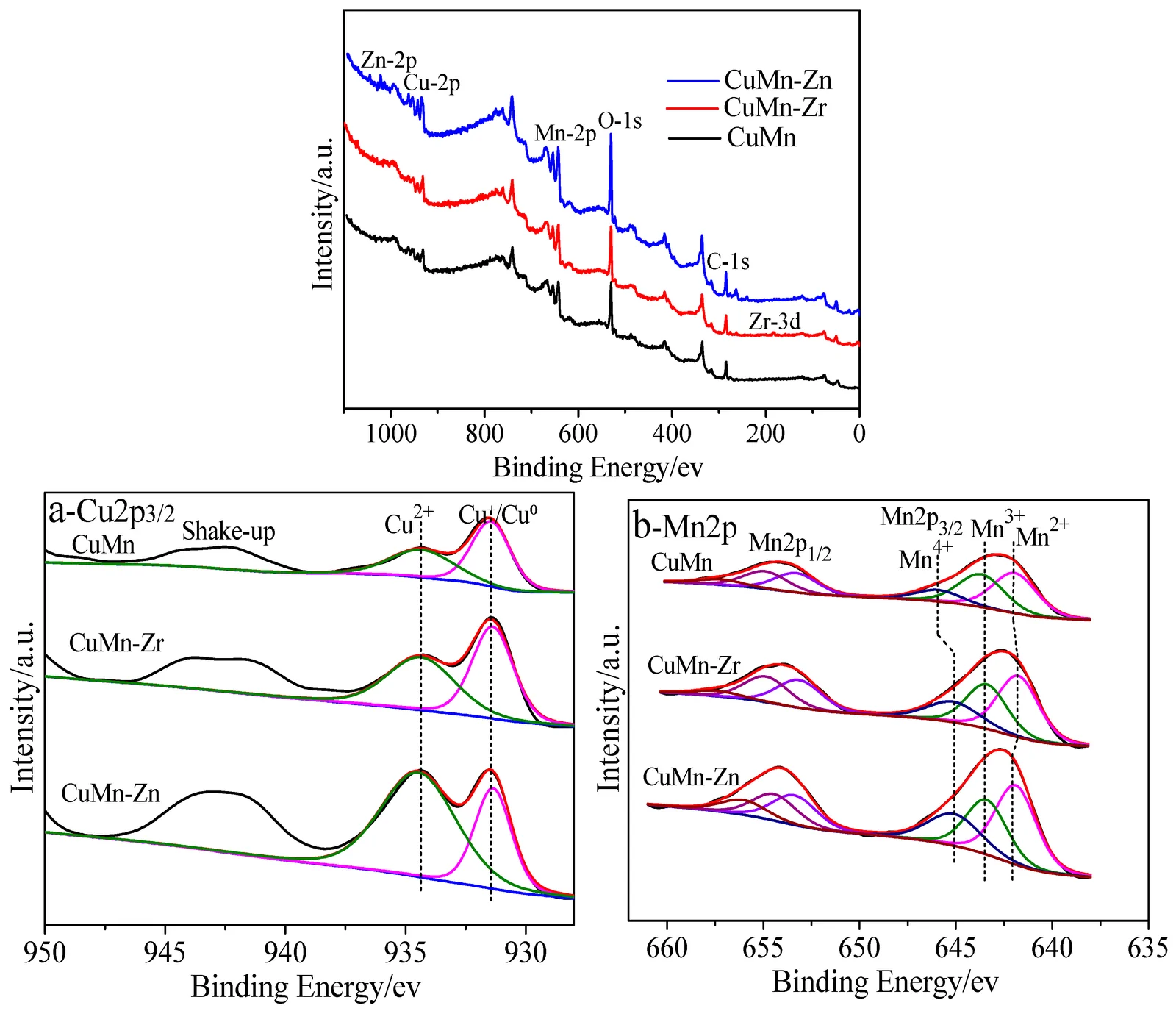
XPS results for representative spent catalysts. **Top**: survey spectra. **Bottom**: (**a**) Cu 2p_3_/_2_ and (**b**) Mn 2p regions. The low-binding-energy Cu component is labeled Cu^+^/Cu^0^. In the survey spectra, black, red, and blue denote CuMn, CuMn-Zr, and CuMn-Zn, respectively. In (**a**), the magenta and green component peaks represent reduced and divalent Cu, respectively. In (**b**), magenta, green, and blue represent the divalent, trivalent, and tetravalent Mn components, respectively; the chemical states are also labeled above the peaks.

For the different-metal series, XPS was performed on spent CuMn as the undoped reference, CuMn-Zr as the doped sample with the highest reported mean CO conversion at 200 °C, and CuMn-Zn as a low-activity comparison. This selection supports representative comparison rather than an exhaustive survey of all dopants. Figure 5 contains the survey spectra and fitted Cu and Mn regions; the numerical results are listed in Table 3 and Table 4. The low-binding-energy Cu component is designated reduced Cu species (Cu^+^/Cu^0^), and its fitted fraction is not a quantitative Cu^+^ fraction alone. The reduced-Cu/Cu^2+^ ratios are approximately 1.43 for CuMn, 1.06 for CuMn-Zr, and 0.47 for CuMn-Zn. Their ordering does not follow the activity ordering: CuMn-Zr is more active than CuMn despite its lower ratio. The XPS component ratio is therefore treated as one surface descriptor rather than as a single-variable explanation of activity.

The adopted Mn 2p fitting model contains Mn^2+^, Mn^3+^, and Mn^4+^ components. The fitted Mn^2+^/Mn^3+^ ratios in Table 4 are approximately 1.19, 1.53, and 1.27 for CuMn, CuMn-Zr, and CuMn-Zn, respectively. CuMn-Zr has the largest ratio among these three tabulated samples. Because the assignments and areas depend on the fitting model, this observation is used as a comparative surface descriptor and does not independently identify the working-state active Mn species.

### 2.2. Effect of Zr Doping Content on Cu-Mn Catalysts

In the metal-dopant comparison, the 5 mol% Zr sample gives the largest reported mean CO conversion at 200 °C under the applied protocol. The nominal Zr content was therefore varied over 0, 2, 5, 8, and 20 mol%. CuMn/Zr-0 denotes the same undoped CuMn reference used in the first series; CuMn/Zr-5 is the same sample as CuMn-Zr.

#### 2.2.1. Catalytic Activity Evaluation

**Figure 6:**
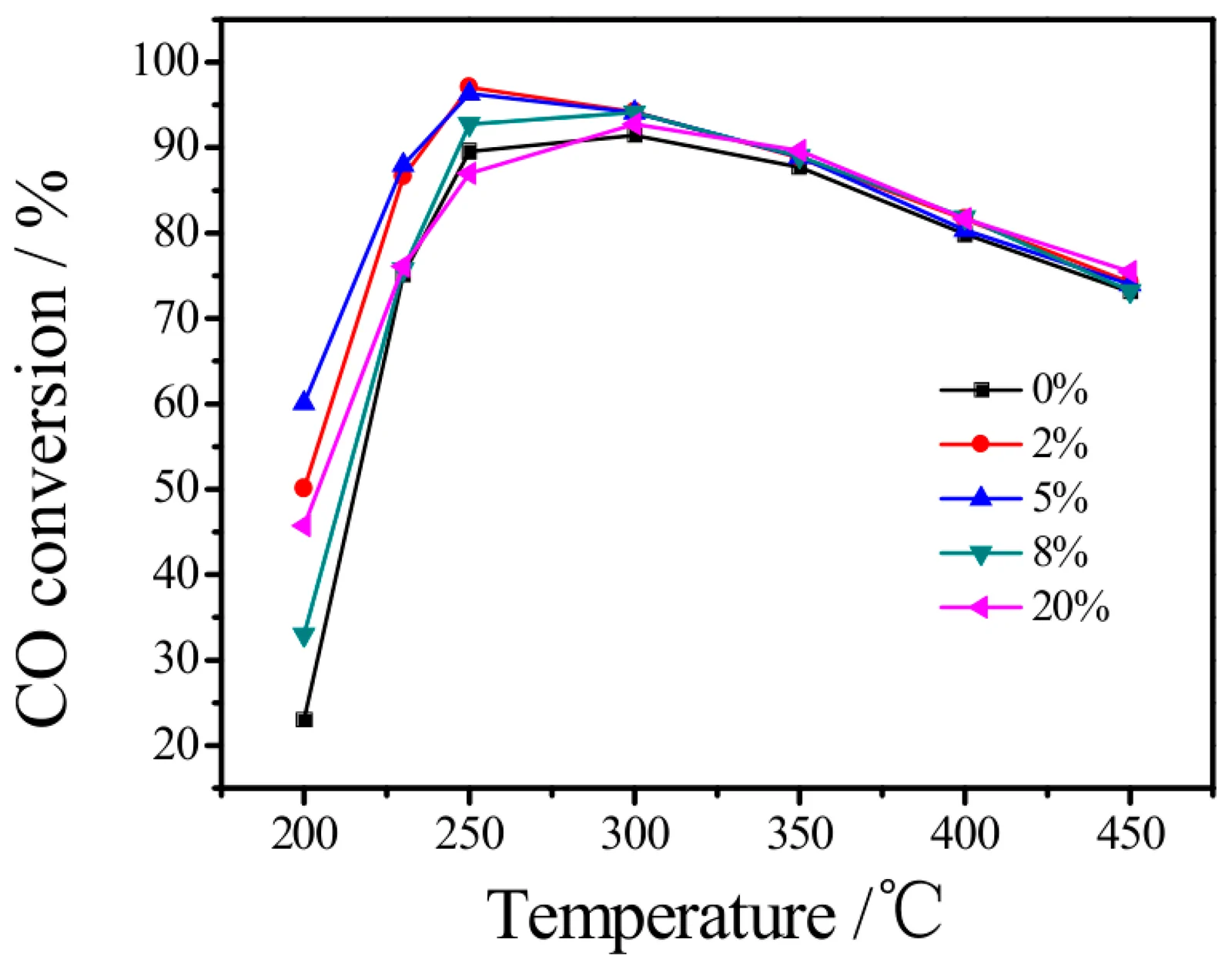
CO conversion of the Zr-loading series during cooling evaluations after the common 450 °C conditioning treatment. The graph is from Hu’s thesis, Figure 3-18(b) [12]. Legend percentages denote nominal metal ion mol%; the intermediate evaluation temperature is 230 °C. The 0% and 5% labels correspond to the shared CuMn and CuMn-Zr samples. The 450 °C conditioning histories are provided in Supplementary Figure S2.

Figure 6 presents the cooling evaluations of Zr series using the activity profiles documented in Hu’s thesis [12]; the preceding 450 °C conditioning histories are provided in Supplementary Figure S2. CuMn/Zr-5 gives the largest reported mean CO conversion at 200 °C, 60.1%. The response is not monotonic with nominal Zr loading, and the 20 mol% sample is more active than the 8 mol% sample at this temperature. Because the individual evaluation values are unavailable, small differences are not interpreted as statistically significant. The 5 mol% composition is the best observed in this tested series under the stated protocol, rather than a statistically established universal optimum.

#### 2.2.2. Characterization of Cu-Mn Catalysts with Different Zr Contents

##### XRD Analysis

**Figure 7:**
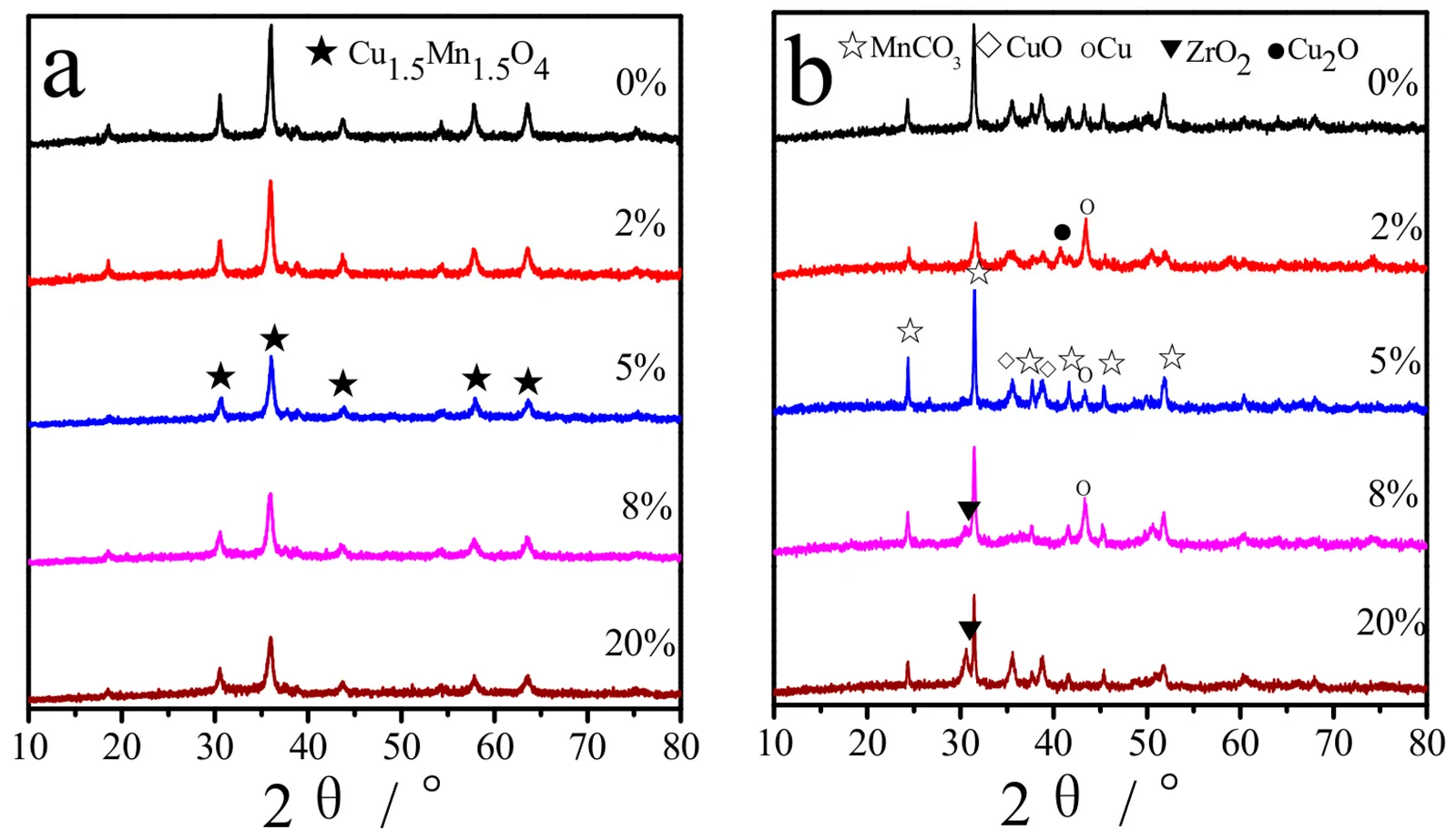
XRD patterns of the Zr-loading series: (**a**) calcined; (**b**) spent. Graphs and phase labels are from Hu’s thesis, Figure 3-19 [12]. Legend percentages denote nominal metal ion mol%.

The calcined Zr-series patterns in Figure 7a show the Cu-Mn spinel-type phase. No separate crystalline Zr-containing phase is resolved in these calcined patterns; a highly dispersed or amorphous component, or an amount below the detection limit, could explain this observation. The spent patterns in Figure 7b contain reflections indexed to MnCO_3_ and Cu-containing phases, including CuO, metallic Cu, and a Cu_2_O-related feature. ZrO_2_-related reflections are identified for both the 8 and 20 mol% samples [12]. Their detection is consistent with a crystalline Zr-containing component after treatment, but does not alone prove pore blockage or explain the activity ordering.

Table 5 lists the phase-specific XRD parameters and BET areas for the Zr series. The (311) spacing of CuMn/Zr-5 is 0.2494 nm, close to the reference value of 0.2495 nm, but this similarity does not prove uniform cation dispersion. The spent Cu sizes are 30.4, 36.1, 50.3, and 24.7 nm for 0, 2, 5, and 8 mol% Zr, respectively. No spent Cu size is reported for the 20 mol% sample. Thus, the 5 mol% sample, which gives the highest reported mean CO conversion at 200 °C, does not have the smallest spent Cu domains. The calcined BET area increases from 43.3 to 57.1 m^2^ g^−1^ across the series, whereas low-temperature conversion does not increase monotonically. BET area alone therefore does not explain the activity maximum.

##### s-TPR Analysis

**Figure 8:**
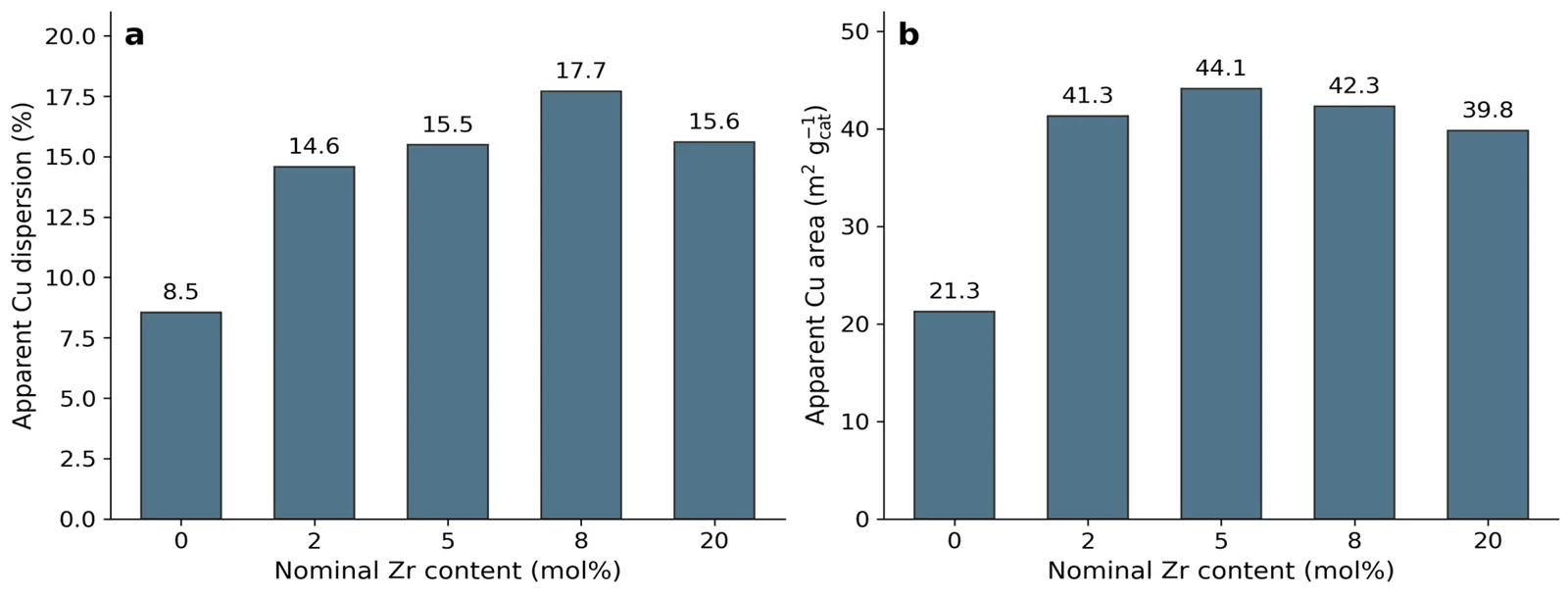
Calculated s-TPR descriptors for the Zr-loading series: (**a**) apparent Cu dispersion; (**b**) apparent Cu surface area per gram of catalyst. Bars use the unrounded calculations from the inputs in Table 6; labels are rounded to one decimal place. They are not new experimental reduction profiles. No error bars are shown because input uncertainties are unavailable.

Table 6 and Figure 8 distinguish apparent fractional Cu dispersion from Cu surface area per unit catalyst mass. The calculated dispersions for 0, 2, 5, 8, and 20 mol% Zr are 8.5, 14.6, 15.5, 17.7, and 15.6%, respectively. The corresponding Cu surface areas are 21.3, 41.3, 44.1, 42.3, and 39.8 m^2^ g^−1^. CuMn/Zr-5 has the largest calculated mass-specific Cu area, whereas CuMn/Zr-8 has the highest apparent fractional dispersion. These different orderings reflect both the reduction-area ratio and Cu mass fraction. The values are model-dependent estimates, and their small differences cannot be assigned statistical significance without uncertainty estimates.

##### XPS Analysis

**Figure 9:**
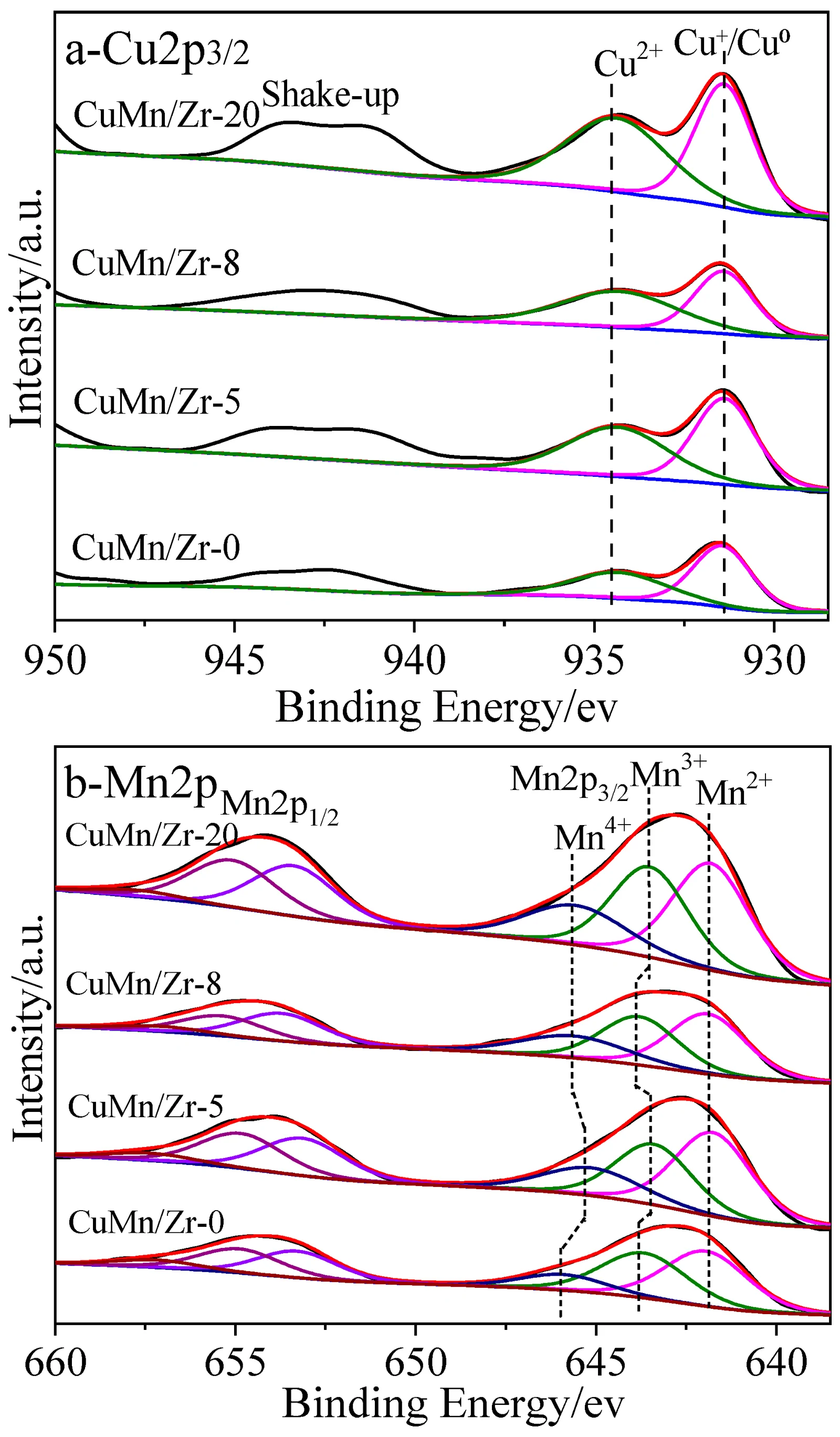
XPS spectra of the Zr-loading series: (**a**) Cu 2p_3_/_2_; (**b**) Mn 2p. CuMn/Zr-0 is the undoped reference; the low-binding-energy Cu component is labeled Cu^+^/Cu^0^.

XPS was performed on spent CuMn, CuMn/Zr-5, CuMn/Zr-8, and CuMn/Zr-20. Table 7 and Table 8 summarize the fitted Cu and Mn components. The low-binding-energy Cu component is consistently described as reduced Cu species (Cu^+^/Cu^0^), including the annotation in Figure 9a. The reduced-Cu/Cu^2+^ ratios are approximately 1.43, 1.06, 0.86, and 0.96, respectively. The lower ratio of CuMn/Zr-5 relative to CuMn shows that its higher reported conversion cannot be explained by a larger reduced-Cu fraction alone. These ex situ fitted quantities are interpreted together with the phase and apparent surface-area descriptors.

The Mn^2+^/Mn^3+^ ratios calculated from Table 8 are 1.19, 1.53, 1.24, and 1.21 for CuMn, CuMn/Zr-5, CuMn/Zr-8, and CuMn/Zr-20, respectively. For CuMn/Zr-20, the fitted fractions are 43.8% Mn^2+^, 36.3% Mn^3+^, and 19.9% Mn^4+^, summing to 100.0%. CuMn/Zr-5 has the largest Mn^2+^/Mn^3+^ ratio among these four samples. This remains a fitting-dependent comparative surface observation and does not establish that Mn^2+^ alone controls WGSR activity.

Across the different-metal series, the Zr-modified sample combines higher CO conversion at 200 °C with a larger model-derived Cu surface area than undoped CuMn. CuMn-Zn gives lower conversion and the lowest BET area in Table 1. The larger spent Cu crystallites of CuMn-Zr relative to CuMn indicate that higher conversion in this comparison is not associated with smaller spent Cu domains.

Within the Zr series, the 5 mol% sample combines the highest reported mean CO conversion at 200 °C with the largest calculated Cu surface area per catalyst mass. Increasing Zr to 8 mol% raises the apparent fractional dispersion but lowers the Cu mass percentage and mass-specific Cu area; the 20 mol% sample also has a lower Cu area. Crystalline ZrO_2_ is detected in both higher-loading spent samples. These observations distinguish fractional dispersion from mass-specific Cu area. Neither the BET area, spent Cu crystallite size, nor reduced-Cu/Cu^2+^ ratio individually follows the activity ranking. The association between calculated Cu area and conversion at 200 °C is descriptive and does not establish a causal structure–activity relationship.

The phase assignments, overlapping reduction features, and ex situ XPS component fractions describe composition-dependent changes in the Cu–Mn samples. These comparative measurements do not independently establish a Cu–Mn synergistic mechanism.

These comparisons apply to the documented conditioning and cooling protocol. The 180 min histories in Supplementary Figures S1 and S2 were recorded near the 450 °C equilibrium conversion and are not long-term durability tests. A dedicated time-on-stream test at 200 °C would be informative for CuMn/Zr-5; the present cooling evaluations do not establish its long-term stability or resistance to sintering. Individual activity values, complete s-TPR integration and Mn-correction records, and some NH_3_ gas parameters are unavailable, limiting statistical comparisons and absolute site quantification. Ex situ measurements describe the sampled material states; resolving the evolving working-state structure requires in situ or operando evidence. The cited electrocatalysis reviews illustrate characterization strategies rather than direct mechanistic evidence for this thermal WGSR system [17,18,19].

## 3. Experimental Procedure

### 3.1. Catalyst Preparation

Cu-Mn catalysts were prepared by co-precipitation using the procedures documented for these samples [12]. CuSO_4_·5H_2_O and MnSO_4_·H_2_O were mixed at a Cu/Mn molar ratio of 1:1, with an initial Cu^2+^ concentration of 0.225 mol L^−1^. Nitrate precursors of Al, Zr, Ce, Fe, or Zn were added to separate portions. The Zr precursor was Zr(NO_3_)_4_·5H_2_O (analytical grade; Tianjin Chemical Reagent Factory No. 3, Tianjin, China). The solutions were maintained at 45 °C and stirred at 300 rpm. Precipitation used 4 mol L^−1^ NaOH, added at a liquid flow rate of 10.5 mL min^−1^ to a total amount of 1.5 times the theoretical requirement. After aging at 45 °C for 30 min, the precipitates were washed to neutrality and dried at 80 °C for 4 h. Calcination was performed in air by heating at 10 °C min^−1^ to 550 °C and holding for 4 h. The undoped sample is denoted CuMn; doped samples are denoted CuMn-M (M = Al, Zr, Ce, Fe, or Zn).

The nominal dopant content is defined as 100n_M_/(n_Cu_ + n_Mn_ + n_M_), where n_M_ is the amount of dopant metal ions and the denominator includes all metal ions, including the dopant. Each catalyst in the metal-dopant comparison contained 5 mol% nominal dopant. The Zr series contained 0, 2, 5, 8, and 20 mol% nominal Zr at a Cu/Mn precursor ratio of 1:1. Table 9 and Table 10 list nominal preparation compositions, rather than independently measured elemental compositions. The same undoped CuMn and 5 mol% Zr-doped catalyst samples were used in both study series. The latter are designated CuMn-Zr in the metal-dopant series and CuMn/Zr-5 in the Zr-loading series. Repeated listing of these samples does not represent additional independent preparations or independent replicate experiments.

Cu and Mn contents were determined by the shared-platform flame atomic absorption spectrometry procedure using a SOLAAR Data Station (Thermo Elemental, Cambridge, UK) with an air–acetylene flame [13]. The Cu contents used for s-TPR calculations are the catalyst mass percentages tabulated for these samples [12]. This mass basis is distinct from the nominal metal ion mole percentages used to specify precursor loading.

### 3.2. Catalytic Activity Evaluation

Activity measurements were performed in a tubular stainless-steel fixed-bed reactor (internal diameter 6 mm) at atmospheric pressure using approximately 0.5 g of catalyst finer than 200 mesh. The dry feed contained 14.50 vol% CO, 16.25 vol% CO_2_, 37.57 vol% H_2_, and 31.68 vol% Ar. The steam/dry-gas molar ratio was 0.6, with dry gas defined as CO + CO_2_ + H_2_ + Ar. The wet-gas flow per catalyst mass was 6400 mL (STP) g^−1^ h^−1^; STP denotes 0 °C and 101.325 kPa. For a 0.5 g loading, this corresponds to approximately 53.3 mL (STP) min^−1^ of wet feed, comprising 33.3 mL (STP) min^−1^ of dry gas and 20.0 mL (STP) min^−1^ of steam, consistent with a liquid-water flow of approximately 0.016 mL min^−1^.

Water was introduced when the reactor reached 190 °C. The catalyst was heated to 450 °C and held for 3 h under the reaction feed before cooling to 200 °C. Both heating and cooling ramps were 15 °C min^−1^. The evaluation temperatures were 450, 400, 350, 300, 250, 230, and 200 °C. At each evaluation temperature, the catalyst was held isothermally for 30 min before GC sampling began; consecutive GC injections were approximately 10 min apart. The feed and effluent were analyzed by GC-TCD (SP3420, Beijing Beifen-Ruili, Beijing, China).

Three activity evaluations were performed at each temperature with the same catalyst loading, and their mean is reported. The catalyst was not reloaded between these evaluations. Individual evaluation results are unavailable, so standard deviations and error bars cannot be reconstructed. The reported means therefore do not establish reproducibility across independent catalyst loadings, and small differences between samples are not assigned statistical significance.

The common 450 °C treatment provides a defined conditioning history before the cooling comparisons. The 30 min isothermal hold specifies the sampling procedure, but is not independent evidence of a strict steady state. High-temperature exposure may produce composition-dependent reconstruction or deactivation. The 180 min conditioning histories are provided in Supplementary Figures S1 and S2. At 450 °C, the measured conversions are close to the calculated WGSR equilibrium reference (75.7%), which limits their sensitivity to changes in catalytic activity. These histories are not dedicated long-term stability tests. The 200 °C results are cooling-stage activity evaluations; no dedicated long-term test at 200 °C was performed.

CO conversion was calculated from inlet and outlet dry-gas CO mole fractions using the documented WGSR expression [12]:

X_CO_ (%) = (y_CO,in_ − y_CO,out_)/[y_CO,in_(1 + y_CO,out_)] × 100.

Here y_CO,in_ = 0.1450, and y_CO,out_ is the outlet CO mole fraction after the removal of water. Mole fractions, rather than numerical percentage values, are inserted into the equation. The factor (1 + y_CO,out_) accounts for the increase in dry-gas molar flow when the WGSR is the only reaction changing that flow. The original individual chromatographic concentrations are unavailable; the source-reported experimental conversion values are retained, with the reporting precision adjusted, rather than regenerated from assumed outlet concentrations.

An ideal-gas WGSR equilibrium reference was calculated for the confirmed feed composition. On a basis of 1 mol dry feed, the inlet amounts are 0.1450 mol CO, 0.1625 mol CO_2_, 0.3757 mol H_2_, 0.3168 mol Ar, and 0.6000 mol H_2_O. Methanation, carbon deposition, condensation, and solid-phase uptake are excluded, so this is a WGSR-only reference rather than a global multiphase equilibrium calculation.

The standard-state equilibrium constant was calculated using NASA7 coefficients for CO, CO_2_, H_2_, and H_2_O in the GRI-Mech 3.0 dataset distributed with Cantera 3.1.0 [20]. The low-temperature sets cover 200–1000 K, with T in kelvin and natural logarithms:

ln Kp = Δa_1_(ln T − 1) + Δa_2_T/2 + Δa_3_T^2^/6 + Δa_4_T^3^/12 + Δa_5_T^4^/20 − Δa_6_/T + Δa_7_

Here Δa_i_ = a_i_,CO_2_ + a_i_,H_2_ − a_i_,CO − a_i_,H_2_O. The coefficient differences are:

(Δa_1_, Δa_2_, Δa_3_, Δa_4_) = (−3.07706939, 1.96119053 × 10^−2^, −3.413893013 × 10^−5^, 2.7197364356 × 10^−8^);

(Δa_5_, Δa_6_, Δa_7_) = (−8.387370829 × 10^−12^, −4652.092173, 7.924685386).

For reaction extent ξ, the equilibrium equation and conversion are:

Kp = (0.1625 + ξ)(0.3757 + ξ)/[(0.1450 − ξ)(0.6000 − ξ)]; X_CO,eq_ (%) = 100ξ/0.1450.

The physical solution satisfies 0 ≤ ξ < 0.1450 mol. Ideal-gas total-pressure terms cancel because the WGSR has zero net change in total gas moles. The calculated equilibrium CO conversions at 200, 230, 250, 300, 350, 400, and 450 °C are 99.0, 98.2, 97.4, 94.4, 89.7, 83.4, and 75.7%, respectively.

### 3.3. Characterization of Catalysts

XRD patterns were collected on a D8 Advance diffractometer (Bruker AXS, Karlsruhe, Germany) using Cu Kα radiation (λ = 0.1541 nm) at 40 kV and 40 mA, over 2θ = 10–80° at 2° min^−1^ [12]. Crystallite sizes were estimated using the Scherrer equation [21]. The tabulated sizes describe coherent diffraction domains of the identified phases. Calcined spinel and spent metallic Cu sizes refer to different phases and cannot be subtracted to quantify Cu crystallite growth.

Textural properties were measured with a QUADRASORB-SI adsorption instrument (Quantachrome Instruments, Boynton Beach, FL, USA). Approximately 0.1–0.5 g of catalyst was placed in the sample tube, evacuated, dried, and degassed at 300 °C for 3 h before N_2_ adsorption–desorption measurements. Specific surface areas were calculated by the Brunauer–Emmett–Teller (BET) method [12]. BET areas and Scherrer crystallite sizes are reported to one decimal place.

H_2_ temperature-programmed reduction (H_2_-TPR), NH_3_ temperature-programmed desorption (NH_3_-TPD), and N_2_O-assisted surface Cu reduction (s-TPR) measurements were carried out using an AutoChem 2920 chemisorption analyzer (Micromeritics, Norcross, GA, USA) equipped with a thermal conductivity detector (TCD).

For H_2_-TPR, 50 mg of catalyst was loaded into a U-shaped quartz reactor at atmospheric pressure. The sample was heated to 120 °C under Ar, purged for 30 min, and cooled to room temperature. The gas was switched to 8.9 vol% H_2_ in Ar at 15 mL min^−1^. After stabilization, the sample was heated from room temperature to 600 °C at 10 °C min^−1^ while the TCD signal was recorded [12].

For NH_3_-TPD, 100 mg of calcined catalyst was subjected to the H_2_-TPR pretreatment. Under Ar, the sample was cooled from 600 to 250 °C at 10 °C min^−1^. NH_3_ was introduced after stabilization, and adsorption was conducted at 250 °C for 100 min. The gas was then switched to Ar and the sample was cooled to 50 °C. After stabilization, the sample was heated to 600 °C at 10 °C min^−1^ with TCD detection [12]. The available source documents do not specify the NH_3_ concentration, the adsorption-gas flow rate, or the duration of the subsequent Ar purge. The profiles are therefore used for qualitative comparison under the documented program, without a calibrated acid-site density.

The N_2_O oxidation–H_2_ reduction procedure (s-TPR) used 50 mg of catalyst. The first reduction followed the H_2_-TPR procedure above. The sample was cooled from 600 to 50 °C, purged with Ar for 30 min, exposed to N_2_O at 20 mL min^−1^ for 30 min at 50 °C, and purged again with Ar for 30 min. For the second reduction, the gas was switched to 8.9 vol% H_2_ in Ar at 15 mL min^−1^ and the sample was heated from 50 to 600 °C at 10 °C min^−1^. The integrated first- and second-reduction signals are denoted A_1_ and A_2_. Under the adopted surface oxidation model, N_2_O + 2Cu → Cu_2_O + N_2_ and subsequent reduction consumes one H_2_ molecule per two oxidized surface Cu atoms [12,22,23].

The apparent fractional Cu dispersion and Cu surface area per unit catalyst mass were calculated separately:

D_app_ = 2A_2_/A_1_;

S_Cu,app_ = D_app_ A_v_ w_Cu_/(100 M_Cu_ n_s_)

D_app_ is a fraction; multiplication by 100 gives the percentage reported in Table 2 and Table 6. w_Cu_ is the Cu mass percentage of the catalyst, A_v_ = 6.02 × 10^23^ mol^−1^, M_Cu_ = 63.5 g mol^−1^, and n_s_ = 1.7 × 10^19^ surface Cu atoms m^−2^. S_Cu,app_ is expressed in m^2^ per gram of catalyst. The area model and inputs are documented in Hu’s thesis [12]. All entries in Table 2 and Table 6 were recalculated with these same constants and the listed A_1_, A_2_, and w_Cu_ values; shared samples use identical inputs in both tables. Source-reported A1, A2, and wCu inputs are retained at their original precision for reproducibility; derived descriptors are rounded only after calculation and reported to one decimal place.

The shared-platform TPR analysis uses Gaussian component fitting and assignments to CuO-related reduction, Cu-Mn solid-solution reduction, and Mn-related reduction [13]. Mn-related features can overlap the Cu-containing reduction regions and can also occur during a second reduction. The available records do not specify the integration limits, baseline treatment, or numerical separation of Mn contributions for the present s-TPR area inputs. Thus, A_1_ and A_2_ are not asserted to be independently validated, element-selective Cu uptake values. D_app_ and S_Cu,app_ are model-dependent estimates; incomplete reduction, nonselective N_2_O oxidation, and overlapping Mn reduction can affect their absolute interpretation [22,23].

XPS (ESCALAB-210, VG Scientific, East Grinstead, UK) was used to examine selected spent catalysts. Mg Kα radiation was operated at 200 W, and the reported base pressure was approximately 10^−8^ Pa. Binding energies were calibrated using the adventitious C 1s signal at 284.6 eV, and spectra were fitted with XPS-PEAK 4.1. The reported fitted fractions describe the adopted peak model and should be distinguished from independently validated absolute surface concentrations [24]. Cu 2p XPS alone does not reliably separate Cu^+^ from Cu^0^; the low-binding-energy component is therefore designated reduced Cu species (Cu^+^/Cu^0^) throughout the text, tables, and Cu XPS figure annotations.

### 3.4. Assistance with Manuscript Preparation and Computational Checks

For this revision, OpenAI ChatGPT (GPT-5.6) in the Work/Codex environment assisted with drafting the scientific text and reviewer responses, English-language revision, reference and consistency checks, analysis-script preparation, and document assembly. Table-derived descriptors and the WGSR equilibrium reference were calculated by executing Python scripts with the source inputs, equations, and constants stated in Section 3.2 and Section 3.3. The tabulated s-TPR descriptors and equilibrium conversions were rechecked at the proof stage using Python 3.12.14. AI was not used to generate missing experimental measurements or new experimental spectra.

## 4. Conclusions

Metal doping produced distinct low-temperature WGSR responses under a common conditioning and cooling protocol. Among the tested compositions, the 5 mol% Zr sample gave the highest reported mean CO conversion at 200 °C, 60.1%, compared with 23.1% for undoped CuMn. Within the Zr series, this sample also had the largest model-derived Cu surface area per catalyst mass, 44.1 m^2^ g^−1^. Its apparent fractional Cu dispersion was 15.5%, whereas the maximum of 17.7% occurred at 8 mol% Zr. Increasing Zr loading did not produce a monotonic activity increase, and higher conversion was not associated with smaller spent Cu crystallites. The results provide a comparative basis for evaluating Zr loading under the tested protocol and demonstrate the importance of distinguishing fractional dispersion from Cu surface area per catalyst mass. Further measurements are needed to test Cu–Mn synergy and to assess long-term stability and resistance to sintering.

## Figures and Tables

**Table 1:** Phase-specific XRD parameters and BET surface areas for the different-metal series.

Sample	2θ (311) °	d (311) nm	Calcined Spinel nm	Spent Cu nm	BET Calcined m^2^ g^−1^	BET Spent m^2^ g^−1^
CuMn	35.92	0.2498	19.2	30.4	43.3	41.6
CuMn-Al	35.94	0.2497	20.4	31.4	48.1	41.0
CuMn-Zr	35.98	0.2494	15.6	50.3	52.1	41.7
CuMn-Ce	36.14	0.2483	19.4	27.7	50.4	36.2
CuMn-Fe	35.88	0.2501	19.6	21.5	48.3	30.9
CuMn-Zn	35.88	0.2501	26.6	35.7	32.0	22.4
Standard	35.97	0.2495	—	—	—	—

Calcined spinel sizes refer to the (311) reflection, and spent Cu sizes to metallic Cu (111). Values follow Hu, Tables 3-4, 3-5, and 3-6 [12]. These different phases do not provide a direct measure of Cu crystallite growth.

**Table 2:** Apparent s-TPR descriptors recalculated for the different-metal series.

Sample	A_1_	A_2_	w_Cu_ %	D_app_ %	S_Cu,app_ m^2^ g^−1^
CuMn	11.11	0.4747	44.60	8.5	21.3
CuMn-Al	11.65	0.8360	54.27	14.4	43.4
CuMn-Zr	10.79	0.8356	51.08	15.5	44.1
CuMn-Ce	10.90	0.7893	45.13	14.5	36.4
CuMn-Fe	9.564	0.6678	46.23	14.0	36.0
CuMn-Zn	12.49	0.6438	56.56	10.3	32.5

A_1_ and A_2_ are the source-reported integrated reduction signals; w_Cu_ is catalyst Cu mass percentage. Inputs are from Hu, Table 3-7 [12], and are retained at source precision for reproducibility. D_app_ and S_Cu,app_ are model-dependent estimates calculated using Section 3.3 and rounded to one decimal place only after calculation.

**Table 3:** XPS fitting results in the Cu 2p_3_/_2_ region for representative spent catalysts.

Sample	Cu^+^/Cu^0^ BE/FWHM eV	Cu^2+^ BE/FWHM eV	Cu^+^/Cu^0^ Fraction %	Cu^2+^ Fraction %
CuMn	931.4 (1.9)	934.3 (3.1)	58.9	41.1
CuMn-Zr	931.3 (2.0)	934.4 (3.3)	51.4	48.6
CuMn-Zn	931.4 (1.8)	934.5 (3.7)	31.9	68.1

Binding energies and FWHM values are in eV; parentheses contain FWHM values. Percentages denote fitted component fractions.

**Table 4:** XPS fitting results in the Mn 2p_3_/_2_ region for representative spent catalysts.

Sample	Mn^2+^ BE/FWHM eV	Mn^3+^ BE/FWHM eV	Mn^4+^ BE/FWHM eV	Mn^2+^ %	Mn^3+^ %	Mn^4+^ %
CuMn	642.0 (2.7)	643.7 (2.8)	645.9 (2.8)	46.7	39.4	13.9
CuMn-Zr	641.8 (2.6)	643.4 (2.5)	645.1 (3.2)	48.2	31.5	20.3
CuMn-Zn	641.9 (2.5)	643.5 (2.5)	645.4 (3.1)	45.2	35.6	19.2

Binding energies and FWHM values are in eV; parentheses contain FWHM values. Percentages denote fitted component fractions.

**Table 5:** Phase-specific XRD parameters and BET areas for the Zr-loading series.

Sample	2θ (311) °	d (311) nm	Calcined Spinel nm	Spent Cu nm	BET Calcined m^2^ g^−1^	BET Spent m^2^ g^−1^
CuMn	35.92	0.2498	19.2	30.4	43.3	41.6
CuMn/Zr-2	35.94	0.2497	15.8	36.1	48.7	46.3
CuMn/Zr-5	35.98	0.2494	15.6	50.3	52.1	41.7
CuMn/Zr-8	35.90	0.2499	17.6	24.7	54.6	42.9
CuMn/Zr-20	35.95	0.2496	16.2	NR	57.1	50.3
Standard	35.97	0.2495	—	—	—	—

Values follow Hu, Tables 3-8, 3-9, and 3-10 [12]. NR means not reported, rather than zero or absence of Cu. The CuMn and CuMn/Zr-5 entries reproduce the shared-sample values in Table 1.

**Table 6:** Apparent s-TPR descriptors recalculated for the Zr-loading series.

Sample	A_1_	A_2_	w_Cu_ %	D_app_ %	S_Cu,app_ m^2^ g^−1^
CuMn	11.11	0.4747	44.60	8.5	21.3
CuMn/Zr-2	10.48	0.7639	50.81	14.6	41.3
CuMn/Zr-5	10.79	0.8356	51.08	15.5	44.1
CuMn/Zr-8	11.12	0.9844	42.84	17.7	42.3
CuMn/Zr-20	10.76	0.8392	45.75	15.6	39.8

Inputs are based on Hu, Table 3-11 [12]. Definitions and constants are identical to Table 2. The shared CuMn-Zr/CuMn/Zr-5 sample uses A_1_ = 10.79, A_2_ = 0.8356, and w_Cu_ = 51.08%, giving S_Cu,app_ = 44.1 m^2^ g^−1^ in both tables. Input precision is retained for reproducibility; derived descriptors are rounded only after calculation.

**Table 7:** Fitted Cu component fractions and ratios for the spent Zr-loading series.

Sample	Cu^+^/Cu^0^ Fraction %	Cu^2+^ Fraction %	(Cu^+^/Cu^0^)/Cu^2+^
CuMn	58.9	41.1	1.43
CuMn/Zr-5	51.4	48.6	1.06
CuMn/Zr-8	46.3	53.7	0.86
CuMn/Zr-20	49.0	51.0	0.96

Fractions are from the adopted Cu 2p fitting model. The CuMn and CuMn/Zr-5 fractions repeat the corresponding shared-sample values in Table 3. The low-binding-energy component does not distinguish Cu^+^ from Cu^0^.

**Table 8:** XPS fitting results in the Mn 2p_3_/_2_ region for the Zr-loading series.

Sample	Mn^2+^ BE/FWHM eV	Mn^3+^ BE/FWHM eV	Mn^4+^ BE/FWHM eV	Mn^2+^ %	Mn^3+^ %	Mn^4+^ %
CuMn	642.0 (2.7)	643.7 (2.8)	645.9 (2.8)	46.7	39.4	13.9
CuMn/Zr-5	641.8 (2.6)	643.4 (2.5)	645.1 (3.2)	48.2	31.5	20.3
CuMn/Zr-8	641.9 (2.5)	643.7 (2.5)	645.7 (3.5)	43.2	34.7	22.1
CuMn/Zr-20	641.9 (2.5)	643.6 (2.5)	645.7 (3.3)	43.8	36.3	19.9

Binding energies and FWHM values are in eV; parentheses contain FWHM values. Percentages denote the fitted fractions of the three Mn components.

**Table 9:** Nominal preparation compositions of the different-metal series.

Sample	Cu Ions mol%	Mn Ions mol%	Dopant Ions mol%
CuMn	50.0	50.0	0.0
CuMn-Al	47.5	47.5	5.0
CuMn-Zr	47.5	47.5	5.0
CuMn-Ce	47.5	47.5	5.0
CuMn-Fe	47.5	47.5	5.0
CuMn-Zn	47.5	47.5	5.0

**Table 10:** Nominal preparation compositions of the Zr-loading series.

Sample	Cu Ions mol%	Mn Ions mol%	Zr Ions mol%
CuMn	50.0	50.0	0.0
CuMn/Zr-2	49.0	49.0	2.0
CuMn/Zr-5	47.5	47.5	5.0
CuMn/Zr-8	46.0	46.0	8.0
CuMn/Zr-20	40.0	40.0	20.0

## Data Availability

The source values used for the tabulated comparisons and the calculated descriptors are provided in this article and its Supplementary Materials, together with the equations and constants used in the calculations. The origins of thesis-derived figures and tables are identified in the corresponding captions and notes and in Hu’s thesis [12]; shared-platform methods are documented in Zhi’s dissertation [13]. The individual chromatographic evaluation values underlying the reported means and complete s-TPR integration, baseline, and numerical Mn-correction records were not available for this revision. These limitations are stated in Section 3.2 and Section 3.3.

## Supplementary Materials

The following supporting information can be downloaded at: https://www.mdpi.com/article/10.3390/molecules31203630/s1. Figure S1: CO conversion during the 450 °C conditioning histories of the different-metal series; Figure S2: CO conversion during the 450 °C conditioning histories of the Zr-loading series.

## References

[1] Cheon B.-S., Kim H.-M., Hwang J.-H., Jeong D.-W. (2025). Highly stable Co/CeO_2_ catalyst for high-temperature water-gas shift reaction using a CeO_2_ support with an optimized precipitant ratio. Fuel.

[2] Zhang J., Jia Y., Jing J., Li W.Y. (2024). Influence of feedstock gas characteristics on hydrogen production via sorption–enhanced water gas shift reaction. Int. J. Hydrogen Energy.

[3] Tabakova T., Ivanov I., Zanella R., Karakirova Y., Sobczak J., Lisowski W., Kaszkur Z., Ilieva L. (2020). Unraveling the effect of alumina-supported Y-doped ceria composition and method of preparation on the WGS activity of gold catalysts. Int. J. Hydrogen Energy.

[4] Tabakova T., Idakiev V., Avgouropoulos G., Papavasiliou J., Manzoli M., Boccuzzi F., Ioannides T. (2013). Highly active copper catalyst for low-temperature water-gas shift reaction prepared via a Cu-Mn spinel oxide precursor. Appl. Catal. A Gen..

[5] Du X.R., Yuan Z.S., Cao L., Zhang C.X., Wang S.D. (2008). Water gas shift reaction over Cu-Mn mixed oxides catalysts: Effects of the third metal. Fuel Process. Technol..

[6] Zhi K.D., Liu Q.S., Zhang Y.G., He S., He R.X. (2010). Effect of precipitator on the texture and activity of copper-manganese mixed oxide catalysts for the water gas shift reaction. J. Fuel Chem. Technol..

[7] He R.X., Liu Q.S., Wu F., Zhi K., Wang N., Zhou C., Liu Q. (2012). Effects of La-doping on the structure and performance of copper-manganese oxides for the water-gas shift reaction. Adv. Mater. Res..

[8] He R., Jiang H., Wu F., Zhi K., Wang N., Zhou C., Liu Q. (2014). Effect of doping rare earth oxide on performance of copper-manganese catalysts for water-gas shift reaction. J. Rare Earths.

[9] Fan Y., Ma J., Yin L., Lin X. (2016). Catalytic performance and characterization of ZrO_2_-modified CuO/Fe_2_O_3_ catalysts in water-gas shift reaction. Ind. Catal..

[10] Djinović P., Levec J., Pintar A. (2008). Effect of structural and acidity/basicity changes of CuO-CeO_2_ catalysts on their activity for water-gas shift reaction. Catal. Today.

[11] Djinović P., Batista J., Pintar A. (2008). Calcination temperature and CuO loading dependence on CuO-CeO_2_ catalyst activity for water-gas shift reaction. Appl. Catal. A Gen..

[12] Hu Q.Q. (2014). Studies on Synthesis, Characteristics and Catalysis Performance of the Metal-doped Copper-Manganese Catalysts for Water-Gas Shift Reaction. Master’s Thesis.

[13] Zhi K.D. (2010). Analysis and Optimization of Reactive Precipitation Processes and Controlled Synthesis of Copper-Based Composite Oxide Water-Gas Shift Catalysts. Ph.D. Thesis.

[14] Hadded A., Massoudi J., Dhahri E., Khirouni K., Costa B.F.O. (2020). Structural, optical and dielectric properties of Cu_1.5_Mn_1.5_O_4_ spinel nanoparticles. RSC Adv..

[15] Zhang Z.C., Zhang Q.X., Yin Y.Q., Wu F., Yu X.F., Xu Y.Z., Li B. (2008). Reducing and catalytic behaviors of copper and manganese oxides water gas shift catalyst. Ind. Catal..

[16] Djinović P., Batista J., Levec J., Pintar A. (2009). Influence of Morphological, Redox and Surface Acidity Properties on WGS Activity of CuO-CeO_2_ Catalysts. J. Chem. Eng. Jpn..

[17] Li J., Gong J. (2020). Operando characterization techniques for electrocatalysis. Energy Environ. Sci..

[18] Xia T., Yang Y., Song Q., Luo M., Xue M., Ostrikov K., Zhao Y., Li F. (2023). In situ characterisation for nanoscale structure–performance studies in electrocatalysis. Nanoscale Horiz..

[19] Zhu Y., Wang J., Chu H., Chu Y.C., Chen H.M. (2020). In Situ/Operando Studies for Designing Next-Generation Electrocatalysts. ACS Energy Lett..

[20] Cantera Developers GRI-Mech 3.0 Thermodynamic Dataset, gri30.Yaml, Distributed with Cantera 3.1.0. NASA7 Coefficient Sets for CO, CO_2_, H_2_, and H_2_O. https://raw.githubusercontent.com/Cantera/cantera/v3.1.0/data/gri30.yaml.

[21] Klug H.P., Alexander L.E. (1974). X-Ray Diffraction Procedures.

[22] Gervasini A., Bennici S. (2005). Dispersion and surface states of copper catalysts by temperature-programmed-reduction of oxidized surfaces (s-TPR). Appl. Catal. A Gen..

[23] Sato S., Takahashi R., Sodesawa T., Yuma K., Obata Y. (2000). Distinction between Surface and Bulk Oxidation of Cu through N_2_O Decomposition. J. Catal..

[24] Shard A.G. (2020). Practical guides for x-ray photoelectron spectroscopy: Quantitative XPS. J. Vac. Sci. Technol. A.

