# Supplementary material for: Effects of Metal Doping on the Structure and Water–Gas Shift Reaction Performance of Copper–Manganese Catalysts
Source: Molecules. 2026 Oct 12;31(20):3630. doi: 10.3390/molecules31203630 (PMC13707856; doi:10.3390/molecules31203630)
Supplement: Supplementary file 1 [file molecules-31-03630-s001.zip › molecules-4537613-supplementary.pdf]

Supplementary Materials

Effects of Metal Doping on the Structure and Water-Gas Shift Reaction Performance of Copper-Manganese Catalysts

Fang Wu, Runxia He*, Qianqian Hu and Gao Saishengtai

*Correspondence: runxiahe@imut.edu.cn

The following figures document the 180 min conditioning histories at 450 °C preceding the cooling-stage evaluations in the main manuscript. At 450 °C, the measured conversions are close to the calculated WGSR equilibrium reference (75.7%), limiting their sensitivity to changes in catalytic activity. These histories are not dedicated long-term stability tests. The experimental protocol is described in Section 2.2 of the main manuscript.

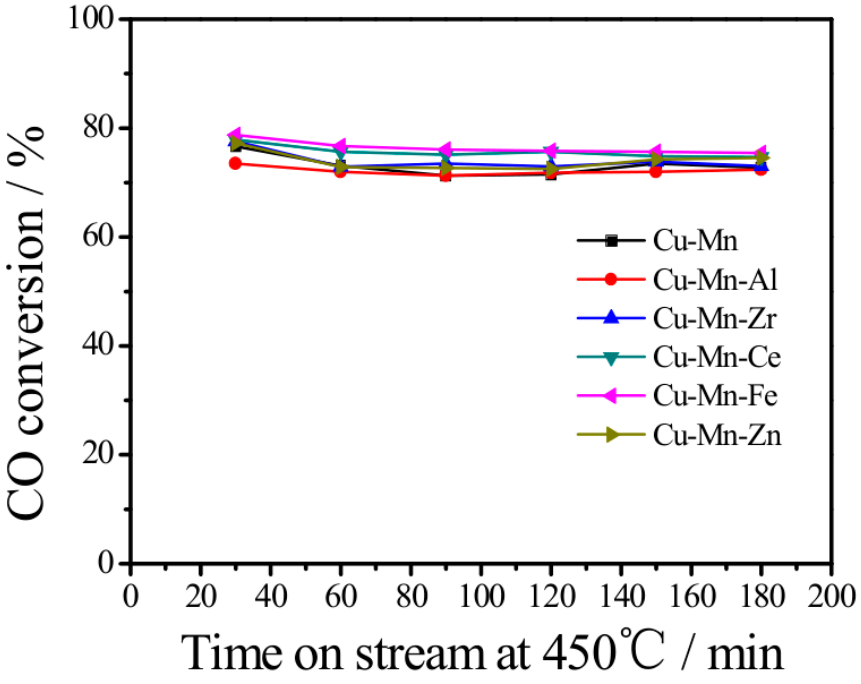

Figure S1. CO conversion during the 450 °C conditioning treatment for the different-metal series. The graph is from Hu, Figure 3-7(a) [12]. Source legends use Cu-Mn for CuMn. All modified samples contain 5 mol% nominal dopant.

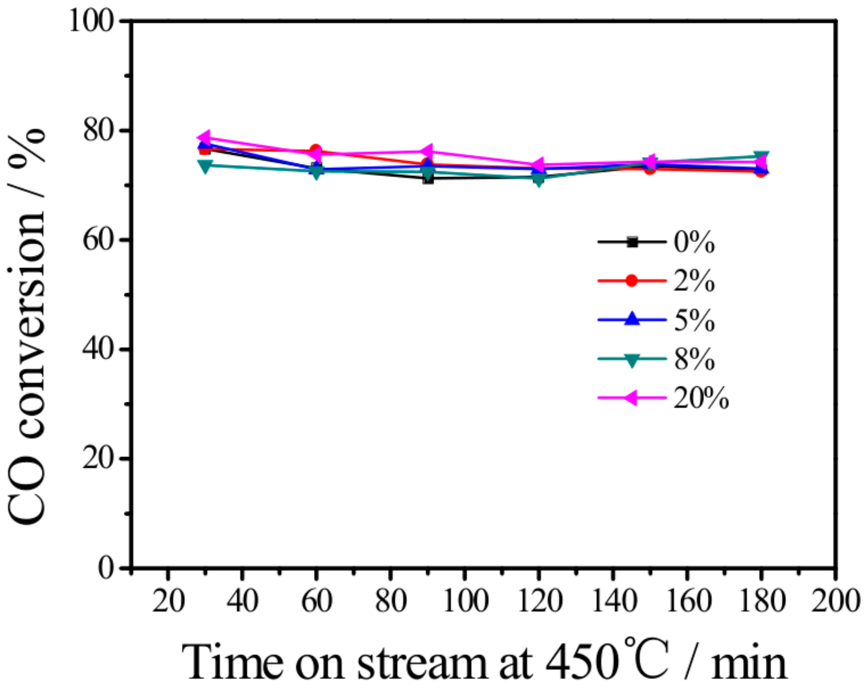

Figure S2. CO conversion during the 450 °C conditioning treatment for the Zr-loading series. The graph is from Hu, Figure 3-18(a) [12]. Legend percentages denote nominal Zr metal-ion mol%. The 0% and 5% labels correspond to the same CuMn and CuMn-Zr samples used in Figure S1.

Reference

[12] Hu, Q. Q. Studies on Synthesis, Characteristics and Catalysis Performance of the Metal-doped Copper-Manganese Catalysts for Water-Gas Shift Reaction. Master's thesis, Inner Mongolia University of Technology, Hohhot, China, June 2014. (in Chinese).
